# Integrated effects of Curcumin application methods and *Trichoderma afroharzianum* on Fusarium wilt management and biochemical responses in hot pepper (*Capsicum annuum*)

**DOI:** 10.1186/s12870-026-08316-0

**Published:** 2026-03-03

**Authors:** Marwa A Zayton, Abdel Wahab M Mahmoud, Karim M Selim, Noura H Mohamed, Shimaa EI Hassanien, Amany M F Attia

**Affiliations:** 1 Department of Plant Pathology, Faculty of Agriculture, Cairo University, Giza, 12613 Egypt; 2 Department of Agricultural Botany, Plant Physiology Division, Faculty of Agriculture, Cairo University, Giza, 12613 Egypt; 3 Department of Botany and Microbiology, Faculty of Science, Al-Azhar University (Girls Branch), Cairo, Nasr City 11754 Egypt

**Keywords:** Biochemical changes, Foliar application, Fusarium wilt, Integrated management

## Abstract

**Background:**

Fusarium wilt caused by *Fusarium oxysporum* is one of the major diseases threatening hot pepper, *Capsicum annuum*, and results in devastating losses. Some plant extracts such as curcumin and turmeric extracts have shown in vitro efficacy against *Fusarium*. These extracts possess broad-spectrum antifungal activity against a number of plant pathogens such as *Fusarium* sp, *Alternaria* sp, and *Botrytis* sp. Curcumin is also known for its safety and low toxicity in plants.

**Result:**

Increasing concentrations of Curcumin progressively inhibited radial fungal growth, reaching a maximum inhibition of 62.2% at 3.0%. In addition, the highest increase in plant growth was recorded with a foliar application of Curcumin in combination with the bio-control agent *Trichoderma afroharzianum* (BCA) concerning the height of the plants and length of roots, the weight of fruit, and also the suppression of disease severity, indicating significant benefits with integrated application approaches. With this, significantly higher concentrations of growth hormones Gibberellic acid (GA3), antioxidant enzymes (Catalase CAT and Peroxidase POD), and micronutrients (Cu, Fe, Zn, B) were found in leaves, while flood (land water addition) treatments tended to have higher Abscisic acid (ABA) levels and enzyme activities related to stress responses. The concentration of secondary metabolites, including capsaicin and total carbohydrates, were higher with combined foliar and BCA treatments over flood application alone, together with an increase in macronutrient uptake (N, P, K, Ca, and Mg).

**Conclusion:**

Results suggest that the combination of Curcumin with *T. afroharzianum* is a non-toxic, effective, and environmentally friendly approach for the control of Fusarium wilt along with improving growth and enhancing biochemical and nutritional potentiality in hot pepper plants. In contrast, flood application of Curcumin showed less or no effect on most of the parameters, which underlines the importance of the mode of application to achieve maximum efficacy.

## Background

The hot peppers (*Capsicum* spp.) have been extensively cultivated not only for their economic importance but also for their vibrant color, distinctive flavor, and valuable nutritional attributes [1]. These fruits are packed with bioactive compounds including capsaicin, carotenoids, flavonoids, vitamins, minerals, and many other beneficial substances [2]. The characteristically spicy aroma of peppers results mainly from the presence and action of capsaicin.

Infection of pepper plants with *Fusarium* sp. results in root rot and stunted growth, which significantly reduces crops. The disease attacks the vascular systems of the plants, and in intensive cases, it results in wilting and plant death. Economic losses due to Fusarium infection can reach up to 50%. To prevent this, it is very important to take some preventive measures; crop rotation to lessen the inoculum in the soil, appropriate sanitation to minimize pathogen spread and also various soil treatment methods and biological fungicides, to help and protect plant roots against *Fusarium* sp [3].

Management of plant disease remains a persistent and constantly evolving challenge that depends on holistic, integrated approaches to achieve sustainable control. Synthetic fungicides effectively suppress Fusarium pepper wilt in the short term but face limitations due to environmental and health risks, pathogen resistance, failure to eradicate persistent soil inoculum, and incompatibility with sustainable integrated pest management (IPM) strategies [4, 5].

Integrated Plant Disease Management IPDM is a strategy utilizing a combination of various strategies, such as cultural, biological, physical, genetic, and chemical methods that contribute most effectively to disease control with very minimal hazards to the environment, economy, and health [6].

Advantages of these integrated approaches are the promotion of plant health, reduction in pesticide residues, protection of nontarget organisms, lowering of environmental contamination, and cost-effectiveness in disease management programs.

As global food demand increases, the implementation of integrated, sustainable disease management practices is fundamental to maintaining crop productivity while protecting ecosystems, ensuring long-term sustainability and resilience in agriculture [7].

*Trichoderma afroharzianum* is considered one of the most useful microorganisms for sustainable agriculture due to previously validated for broad-spectrum antagonism against *Fusarium solani*,* Macrophomina phaseolina*,* Rhizoctonia solani*,* and Sclerotium rolfsii*, and its importance in the mechanisms of plant growth promotion [8, 9]. *Trichoderma* species have a well-documented capability for enhancing plant growth and diminishing disease incidence. It secretes degradative enzymes, including chitinase, glucanase, and carboxymethyl cellulase, which in turn cause hydrolysis of the pathogenic fungi cellular walls, hindering their proliferation [8, 9].

On the other hand, Curcumin is a phenolic compound and a major active constituent of the rhizomes of turmeric (*Curcuma longa*), which has been suggested to possess antioxidant and anti-inflammatory activities and activate plant defense enzymes like catalase and superoxide dismutase [10]. It evokes systemic defense reactions in plants against disease [11]. It increases soil fertility by making phosphorus [12] and iron available for uptake, thus enhancing root growth and water and nutrient absorption.

Studies have shown that *Trichoderma afroharzianum* can function as a biochemical alternative to pesticides and offers environmentally friendly protection while having negligible adverse effects on soil and ecosystems [13]. This fungus has also been reported to stimulate the production of plant growth hormones [14], which enhances yields and improves the quality of crops.

This research has been conducted to investigate the efficacy of applying Curcumin through different methods in combination with *T. afroharzianum* for the management of Fusarium wilt and its effect on biochemical changes in hot pepper.

## Materials and methods

### Source of Curcumin

Curcumin is sourced from the rhizomes of the turmeric plant, and the Curcumin (90%) used in this research was obtained from the Elabge Factory for Extraction of Vegetables and Essential Oils, Egypt.

### Source of bio-agent

*Trichoderma afroharzianum* was previously isolated and molecularly identified, with the sequences submitted to the GenBank database under accession numbers PQ302270 for the Internal Transcribed Spacer (ITS) region and PQ389509 for the Translational Elongation Factor 1-α (Tef1-α) gene [9].

### Source of plant

A one-month-old hybrid Omega pepper seedlings, sourced from commercial nurseries, were used for conducting the research experiments.

### Isolation, purification, and pathogenicity tests of the pathogen

Samples of pepper plants (hybrid Omega) exhibiting symptoms of wilting, chlorosis, stunting, and plant death were collected from the greenhouse of the Plant Pathology Department, Cairo University. Infected plants’ root systems were thoroughly washed with water, and small segments were taken from the crown and stem regions. These plant tissues were surface-sterilized in 0.1% sodium hypochlorite for 1 min., followed by triple rinsing with sterile distilled water. The sterilized segments were then air-dried on sterile filter paper and aseptically transferred onto Potato Dextrose Agar (PDA) medium, incubated at 25^◦^C. pure cultures of *Fusarium* spp. were obtained through subculturing of the fungal colonies, and microscopic examination was performed for identification purposes. The pure isolates were preserved for subsequent experiments [15].

For pathogenicity test, sterile distilled water (around 10 mL) is added to the surface of the 7days old *Fusarium oxysporum* culture plate. The fungal mycelium is gently rubbed with a sterile glass to release the conidia into the water, creating a spore suspension.

Using a hemocytometer, the spore concentration is adjusted to 1 × 10^6^ spores/mL.

The roots of one-month-old hybrid Omega pepper seedlings prepared in trays are dipped in the spore suspension for 10–15 min, then the seedlings are planted in pots (20 cm individually). Inoculated seedlings are maintained under optimal temperature and humidity conditions suitable for disease development [15]. Plants are regularly observed for symptoms such as wilting, chlorosis, and stunting. To further verify the pathogenesity of the isolate, reisolation of the pathogen from diseased plants were performed according to the Koch’spostulates.

### Disease assessment

Fusarium wilt severity was assessed and rated using the disease severity scale described by Kabeto [16].

### Influence of different concentrations of Curcumin on the linear growth of *Fusarium oxysporum*

A food poisoning method was used where Curcumin powder was mixed with dissolved PDA medium up to final concentrations (0.5, 1, 1.5, 2, 2.5, 3%), each concentration was set up with three parallel repetitions, and the medium with no Curcumin was used as the control group. All the treatments and controls were inoculated with a mycelium plug (in diameter 4 mm) of *Fusarium oxysporum* in the center of the plate. When the colonies of the control group completely covered the plate, the colony diameter of each plate was measured, and the inhibition rate (IR) was calculated by the following formula: IR (%) = ((Dc-Dt)/Dc)*100; where IR represents the inhibition rate, Dc represents the colony diameter of the control group, and Dt represents the colony diameter of the Curcumin treated group [17].

### Influence of Curcumin against Fusarium wilt in hot pepper

One-month-old hybrid Omega pepper seedlings were dipped in a *Fusarium oxysporum* spore suspension (1 × 10^6^ spores/mL) for 15 min. and individually planted in pots 25 cm contain a sterilized clay-sand mixture (1:1 w: w) [18]. Curcumin was applied either by spraying, flooding (land water addition), or an alternating regimen of spraying and flooding. Plants were treated four times at15 day intervals. Disease was assessed as mentioned before.

### Influence of Curcumin combined with Trichoderma afroharzianum against *Fusarium oxysporum* in hot pepper

In the present experiment, a similar design was employed in the previous experiment, with the addition of mixing *T. afroharzianum* inoculum (cultured on a sand-barley medium (1:3 w: w) for 15 days at 25 ± 2 °C) at a rate of 3% (w/w) into the sterilized clay-sand mixture prior to planting the pepper seedlings [19, 20].

### Influence of Curcumin application on the biochemical changes in hot pepper

Pepper leaves were collected 45 days after transplanting from plants treated with different methods of Curcumin application, ensuring sampling at the same leaf growth stage. Plants grown in soil infested with *T*. *afroharzianum* but without Curcumin application served as the bio-control agent (BCA) control. Meanwhile, plants grown without Curcumin application in non-infested soil served as the untreated control.

The samples of pepper leaves were dehydrated in an electric oven at70°C for 24 h, and then 0.2 g of plant material was digested by adding 5 mL of concentrated sulfuric acid and heating for 10 min until it reached 100 °C, followed by the addition of 0.5 mL of perchloric acid with continuous heating to 350 °C until a clear solution was obtained [21, 22].

The total nitrogen (N) content of the dried leaves was determined using the modified micro Kjeldahl method as outlined by Helrich [23]. phosphorus (P) was quantified colorimetrically utilizing the chloro-stannous molybdophosphoric blue color technique in sulfuric acid as per referred by Jackson 1973 [21], the concentrations of potassium(K), and calcium (Ca) were measured using a flame photometer apparatus (CORNING M 410, Germany), and the concentrations of Cu, Fe, Mg, Zn, Mn and B were measured using the chlorostannous molybdophosphoric acid blue color technique in sulfuric acid, An atomic absorption spectrophotometer with air acetylene and fuel (PyeUnicam, model SP-1900, US) was used to measure the concentrations of magnesium, zinc, manganese, copper, and iron. The phosphomolybdic acid technique was used to determine the total amount of carbohydrates in the leaf samples [23].

Capsaicin and dihydrocapsaicin standards were purchased from Fluka Chemical Co. (Buchs, Switzerland). All solvents used as mobile phase were of HPLC grade and supplied by Aldrich (Steinheim, Germany). All samples were first dried, and then extracted using the method of Collins [24] with slight modifications. For capsaicinoid extraction, each dried pepper sample (5 g) was placed in ethanol (5 mL) in a 120 mL glass bottle equipped with a Teflon lined lid. Bottles were capped and placed in a water bath at 80 °C for 4 h, then swirled manually every hour. Samples were removed from the water bath and cooled to room temperature. The supernatant layer of each sample (5 mL) was filtered through 0.45 μm filter paper into a HPLC sample vial using a 5 mL disposable syringe (Millipore, Bedford, MA, USA). The vial was capped and stored at 5 °C in a refrigerator until analysis.

The major capsaicinoids in peppers, capsaicin and dihydrocapsaicin, were determined by comparison to external reference standards injected under the same conditions. Their identification was based on the retention times measured under identical HPLC conditions while their quantitative determination in the different peppers samples was carried out using the peak areas. The ratio between these capsaicinoids was calculated by dividing capsaicin and dihydrocapsaicin contents to the total capsaicinoids [25].

### Influence of Curcumin application on the endogenous phytohormones

Gibberellic acid (GA3) and Abscisic acid (ABA) determinations were conducted as per Fales [26], according to the procedure outlined by Furniss [27], freeze-dried plant samples (equivalent to 6 g FW) were ground to a fine powder using a mortar and pestle in order to quantify the endogenous phyto-hormones using Ati-Unicum gas, liquid chromatography, 610 Series, equipped with a flame ionization detector. Three extractions of the powdered material were made (once for three hours and twice for one hour). Under dark conditions at 4 °C with methanol (80% v/v, 15 mL/g FW) and butylated hydroxy toluene (2, 6-di-tert-butyl-p-cresol) were added as an antioxidant. At 4000 rpm, the extract was centrifuged. After transferring the supernatant into aluminum foil-wrapped flasks, the residue was extracted twice. After combining the supernatants, the volume was lowered to 10 mL while being vacuumed at 35 °C. After bringing the aqueous extract’s pH down to 8.6, it was extracted three times using the same amount of pure ethyl acetate. After being dehydrated over anhydrous sodium sulfate, the mixed alkaline ethyl acetate extract was filtered. After being vacuum-evaporated to dryness at 35 °C, the filtrate was re-dissolved in 1 mL of absolute methanol.

The samples that were produced, using the Polle et al. method [28] had their catalase (CAT) activity measured. To create a homogenized mixture, 0.5 g of fresh sample was mixed with 5 mL potassium phosphate buffer, 0.5% Triton X-100, 2% N-vinylpyrrolidone (NVP), 5 mM ethylene-diamine-tetra-acetic acid (EDTA), di sodium salt dihydrate, and 1 mM ascorbic acid. The mixture was then ground using liquid nitrogen. The activated enzymes were measured from the supernatants after the samples were centrifuged for 25 min at 4 °C at 1000 rpm [29, 30]. The CAT values were expressed in units of milligrams per protein.

Determinations of peroxidase using the procedure suggested by [31, 32], which involved freezing (0.5 g) in liquid nitrogen to extract the peroxidase enzyme, the quantity of peroxidase was calculated. 10 mL of extraction solution (50 mM phosphate buffer, pH 7), including 0.5 mM EDTA and 2% PVPP (w/v), were used to grind the samples before they were centrifuged for 20 min at 3930 rpm. Using a spectrophotometric technique, the peroxidase activity was measured in the ensuing supernatant by forming guaiacol in a l mL reaction mixture (450 µl 25 mM guaiacol, 450 µl 225 mM H_2_O_2_) and 100 µl crude enzymes.

### Statistical analysis

The collected data were analyzed using R Statistical Software (version 4.4.2; [33]). An analysis of variance (ANOVA) was conducted, and mean differences among treatments were assessed using Tukey’s multiple range test with a significance level of α = 0.05.

## Results

### Isolation, purification, and pathogenicity tests of the pathogen

The fungus was isolated from wilting, chlorotic, and stunted pepper plants. Identification was done based on cultural characteristics, growth patterns, and microscopic morphological features of hyphae and spores. Accordingly, the pathogen was identified as *Fusarium oxysporum* (Schlecht), and pathogenicity tests confirmed that it is *Fusarium oxysporum f. sp. Capsica.*

### Effect of different concentrations of Curcumin on the linear growth of *Fusarium oxysporum*

**Fig. 1:**
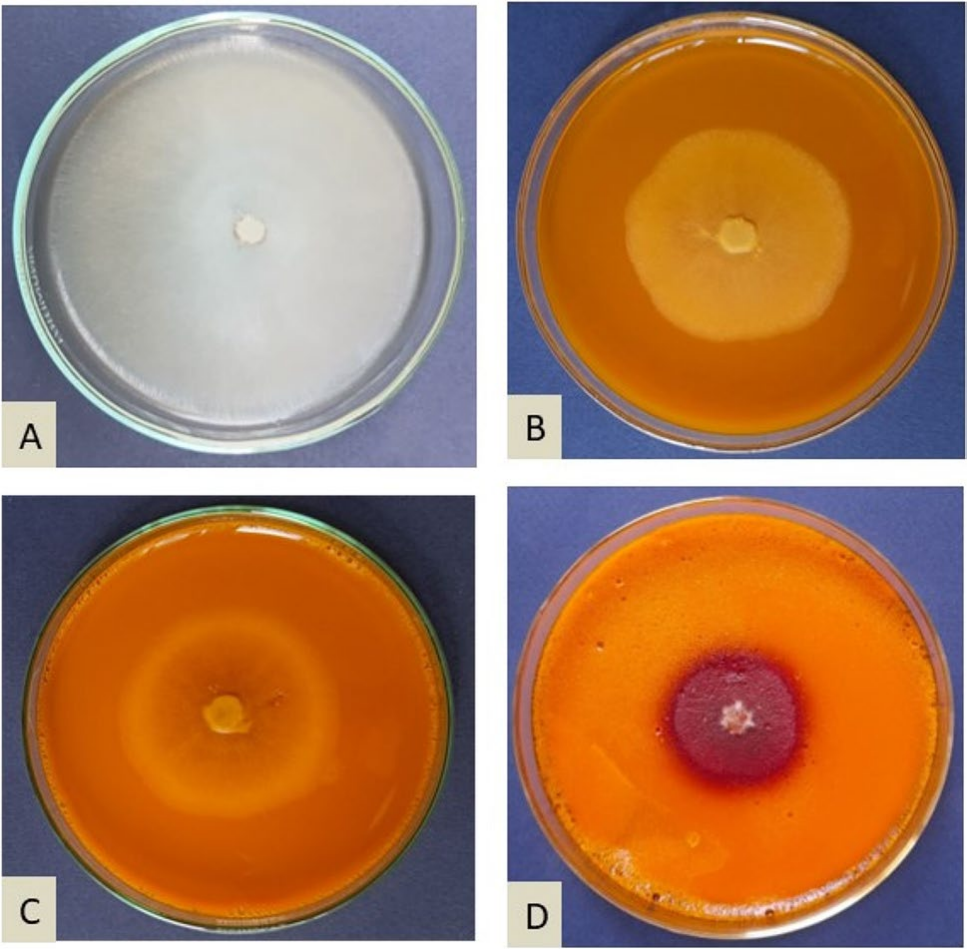
Effect of Curcumin concentrations on the linear growth of *F. oxysporum*, **A** control, **B** at1%, **C** at 2% and **D** at 3% curcumin conc

Data (Table 1) clearly show the dose-dependent antifungal activity of Curcumin against *F. oxysporum*, as evidenced by the progressive inhibition of radial fungal growth with increased concentrations of Curcumin. The lowest concentration, 0.5%, recorded a radial growth of 68.0 mm, which represents 24.4% inhibition in comparison to the control value, 90.0 mm. Inhibition rates were increased correspondingly to 43.3 and 50.0% at concentrations of 1.0 and 1.5%, respectively, with a corresponding reduction in radial growth. As the concentration increased to 1.0 and 1.5%, inhibition rates were increased correspondingly to 43.3 and 50.0%, respectively, with a reduction in radial growth. As expected, the highest concentration of Curcumin studied in this experiment, 3.0%, resulted in the highest inhibition, reaching 62.2%, with a drastic fall of fungal growth to only 34.0 mm (Fig. 1).

**Table 1:** Effect of different concentrations of Curcumin on the linear growth of *Fusarium oxysporum*

Curcumin Conc.%	*Fusarium oxysporum*	
	Linear growth, mm	% Inhibition
0.5	68.0^b^	24.4
1.0	51.0^bc^	43.3
1.5	45.0^c^	50.0
2.0	39.0 ^c^	56.7
2.5	36.5 ^c^	59.4
3.0	34.0 ^c^	62.2
Control	90.0^a^	---

Statistically significant differences between means were indicated by different letters (s) according to Tukey’s multiple range test at α = 0.05

### Effect of different applications of Curcumin on the plant growth parameters, fruit weight, disease severity, and control efficacy in pepper plants

Data presented in Table 2 demonstrate the effects of various Curcumin (Cur) applications (foliar spray, flooding (land water addition) and alternating application of spraying and flooding with Curcumin) in the presence of the biological control agent *T*. *afroharzianum* (BCA) on plant growth parameters, fruit weight, disease severity, and control efficacy in pepper plants.

**Table 2:** Effect of Curcumin application methods combined with the biological control agent on plant height and root length and disease severity

Treatments	Plant height (cm)	% Inc.	Root length (cm)	% Inc.	Average of fruits weight (g)	% Inc.	% Disease severity	% Efficacy
Cur+ Spray	35.16^b^	36.80	36.67^b^	27.87	52.30^c^	40.21	30.00^de^	55.02
Cur+ Flood	29.67^de^	15.44	32.83^cd^	14.28	33.60^h^	-9.91	40.00^b^	40.03
Cur + mix	31.00^cd^	20.62	35.50^bc^	23.69	42.90^e^	15.01	36.67^bc^	44.97
BCA + Cur+ Spray	38.83^a^	51.08	40.16^a^	40.06	64.40^a^	72.65	23.33^f^	65.06
BCA + Cur+ Flood	33.67^bc^	31.01	34.40^bc^	19.86	44.90^d^	20.37	33.33^cd^	50.07
BCA + Cur+ mix	35.50^bc^	38.13	36.50^b^	27.17	57.90^b^	55.22	26.67^ef^	59.97
Control + BCA	27.00^ef^	---	30.83^de^	---	40.50^f^	---	40.00^b^	---
Control	25.70^f^	---	28.70^e^	---	37.30^g^	---	66.67^a^	---

Curcumin (Cur) application methods either by spraying (Cur+spray), flooding (Cur+Flood) or an alternating regimen of spraying and flooding (Cur + mix). These same application methods were also tested in combination with the biocontrol agent (BCA)

Statistically significant differences between means were indicated by different letters (s) according to Tukey’s multiple range test at α = 0.05

Among the treatments, the combined application of Curcumin applied via foliar spray in the presence of BCA consistently produced the most significant enhancements across all measured parameters. This treatment achieved the greatest increase in plant height (38.83 cm, + 51.08%), root length (40.16 cm, + 40.06%), and average fruit weight (64.40 g, + 72.65%) compared to the untreated control. Correspondingly, it also resulted in the lowest disease severity (23.33%) and the highest efficacy in disease control (65.06%).

The alternating application of spraying and flooding with Curcumin in the presence of BCA also showed considerable improvement in growth (plant height of 35.50 cm, + 38.13%; root length of 36.50 cm, + 27.17%) and fruit weight (57.90 g, + 55.22%) with a substantial reduction in disease severity (26.67%) and efficacy (59.97%).

Conversely, Curcumin applied alone by flooding resulted in the least favorable outcomes among treated groups, with relatively low increases in growth parameters (plant height + 15.44%, root length + 14.28%) and a decline in fruit weight (-9.91%), coupled with higher disease severity (40.00%) and reduced the efficacy of disease control (40.03%).

### Effect of different Curcumin application methods on hormones and enzymatic activities

**Fig. 2:**
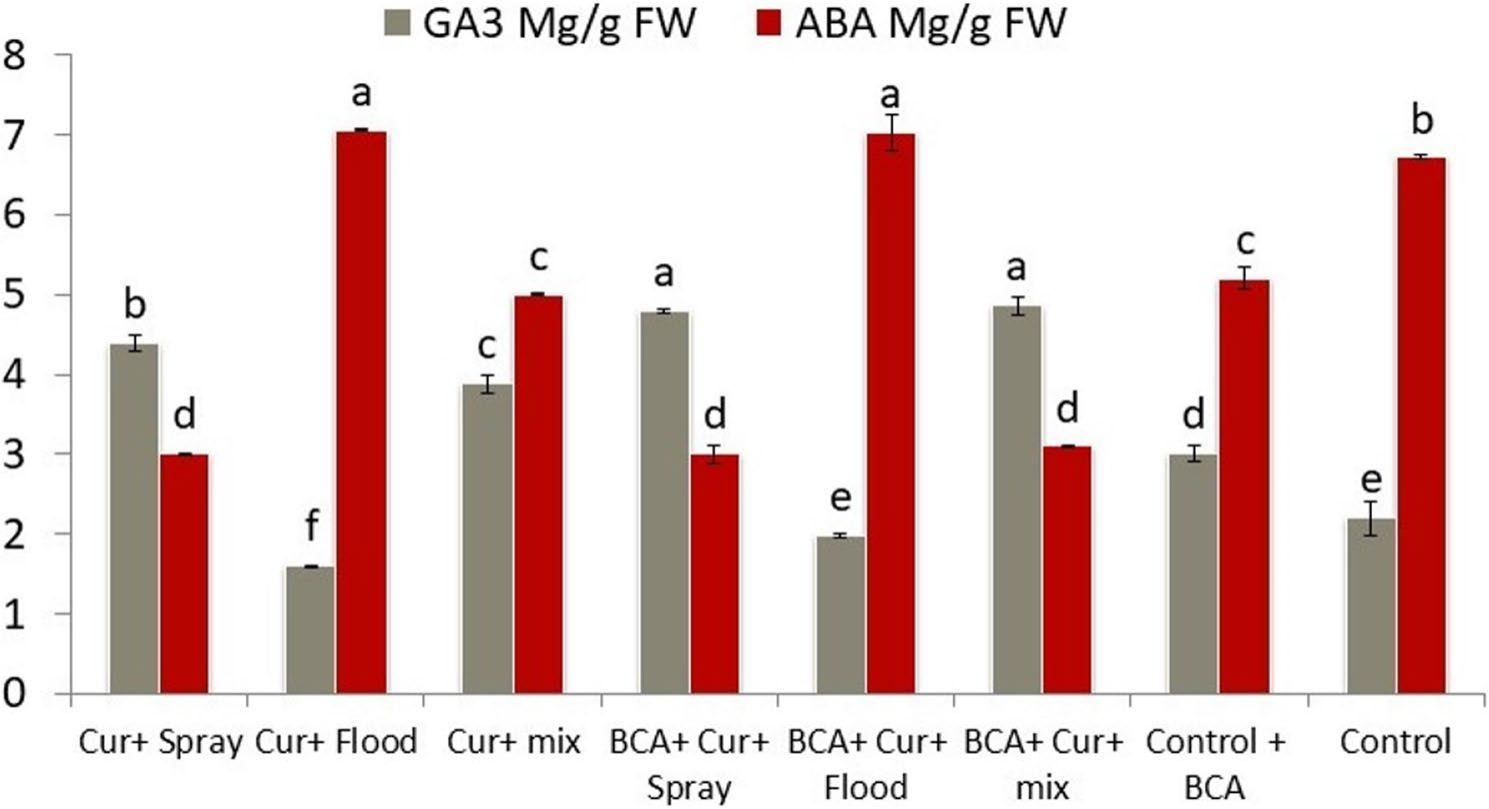
Effect of Curcumin application methods combined with the biological control agent on Gibberellic acid (GA3) and Abscisic acid (ABA) hormones

**Fig. 3:**
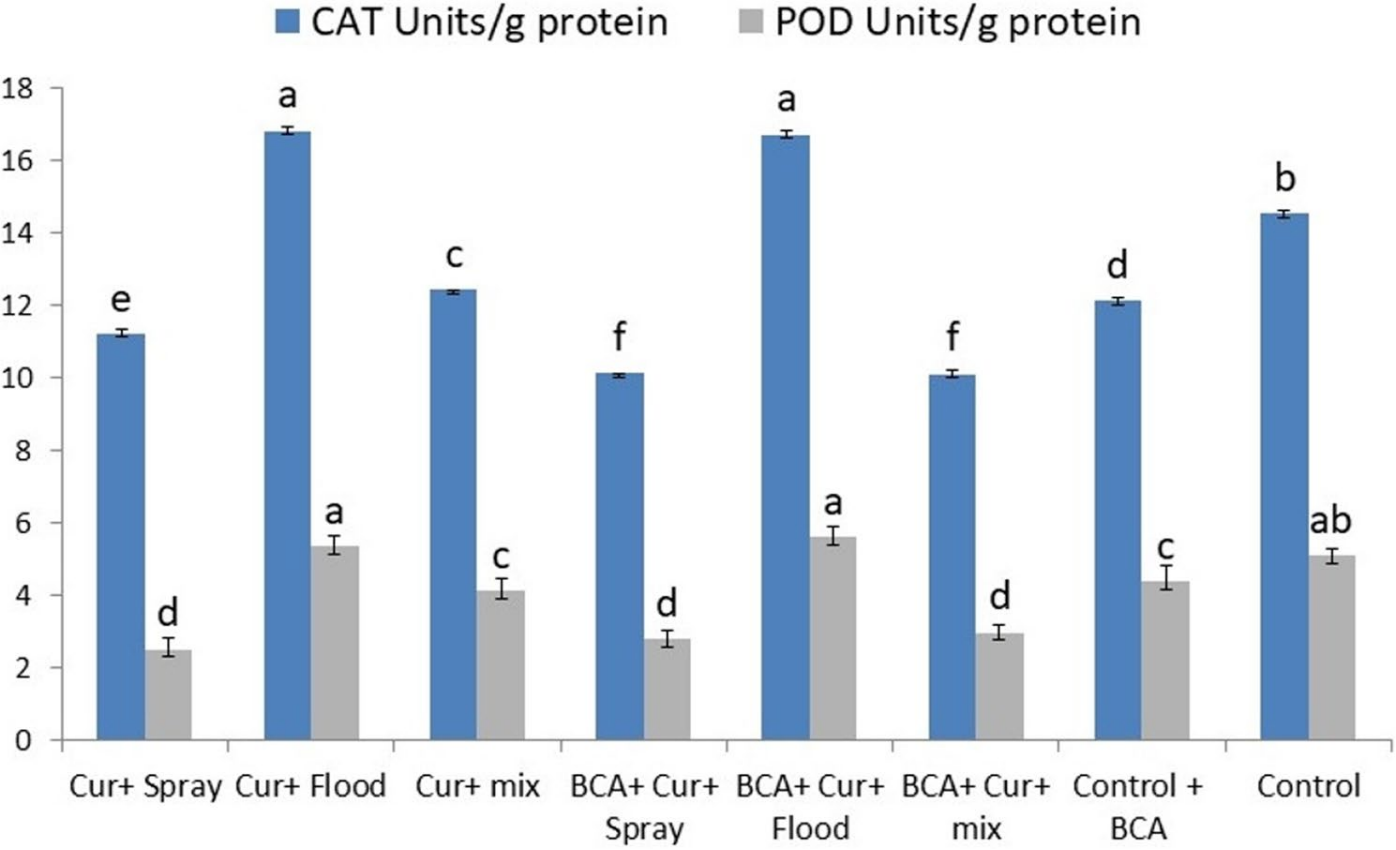
Effect of Curcumin application methods combined with the biological control agent on Catalase (CAT) and Peroxidase (POD) enzymatic activities

Figures 2 and 3 show how different treatments of Curcumin application with or without BCA affected the levels of hormones (GA3 and ABA) and enzymatic activities (CAT and POD) in pepper leaves. The data are presented as absolute concentrations or activities together with their percentage changes from the control. GA3 level was very much elevated due to Curcumin spraying up to 4.399 µg/g FW (+ 99.9%) as compared to 2.2 µg/g FW in control. When Curcumin spray was applied in the presence of BCA, increased GA3 levels to 4.796 µg/g FW (+ 118%), indicating the synergistic effect. The alternation of spraying and flooding also increased GA3, especially when BCA was present (4.862 µg/g FW; +121%), showing the highest GA3 levels among treatments. On the other hand, flood application of Curcumin resulted in a decrease in GA3 to 1.601 µg/g FW (-27.2%), and even with BCA, this effect was only slightly alleviated (1.975 µg/g FW; -10.2%) (Fig. 2).

ABA levels were the highest in flood experiments (7.058 µg/g FW; +5.0% with Curcumin only, and 7.036 µg/g FW; +4.7% with BCA). All spraying treatments with or without BCA that were tested in this experiment consistently lowered the level of ABA compared to the control (e.g., 3.010–3.100 µg/g FW, approximately − 53 to -55%), respectively. The alternating treatments resulted in intermediate ABA values but were still significantly lower than the control (Fig. 2).

In comparison to the control (14.517), the greatest CAT activity was found in flood situations that featured Curcumin alone (16.806; +15.76%) and also with BCA (16.699; +15.0%). Spray and alternating treatments, irrespective of BCA presence, had lower CAT activities that ranged from 10.098 to 12.409 units/g protein, indicating that CAT activities were reduced approximately within 14–30%.

The behavior of POD activity is the same as that of CAT with the highest enzyme activities found in flooded treatments: 5.369 (+ 5.2%) for Curcumin only, and 5.612 (+ 9.9%) with BCA. The activity of POD was drastically reduced in the sprayed and alternated leaves and dropped down to 2.5–3.0 units/g protein (up to -50% relative to control’s 5.104) (Fig. 3).

### Effect of different application methods of Curcumin on the micronutrient concentrations in pepper leaves

Data presented in Table​‍​‌‍​‍‌​‍​‌‍​‍‌ 3 display the Influence of different Curcumin (Cur) applied methods (spray, flood, or alternating spray and flood) in the presence of the biological control agent (BCA) on the levels of micronutrients (Cu, Fe, Mn, Zn, and B) in pepper leaves. Among all treatments, the foliar application of Curcumin in the presence of the biological control agent (BCA) was the most effective in terms of leaf concentrations of copper (10.62 ppm), iron (117.73 ppm), zinc (134.11 ppm), and boron (48.66 ppm). In these treatments, the measured elements registered a substantial increase over the control: copper (+ 31.93%), iron (+ 52.26%), zinc (+ 71.65%), and boron (+ 65.12%). This indicates that the combination of BCA with foliar Curcumin application substantially promotes micronutrient uptake, which can be explained, for instance, by healthier roots, increased foliar absorption, or enhanced stress tolerance. In the same way, only the foliar application of Curcumin demonstrated good results, reaching the second-highest levels of Cu, Fe, and Zn, with an increase ranging from 29.33 (Cu) to 57.26% (Zn). Nevertheless, the use of Curcumin solution by flooding generally yielded lower results or even negative efficacy, especially in the case of iron and zinc uptake. At the same time, Curcumin application via flooding in the presence of BCA, resulted in an increase of 10.74% for Fe and 11.24% for Zn.

**Table 3:** Effect of Curcumin application methods combined with the biological control agent on micronutrient elements in pepper leaves

Treatments	Concentration of elements in leaves (ppm)									
	Cu	% Inc.	Fe	% Inc.	Mn	% Inc.	Zn	% Inc.	B	% Inc.
Cur+ Spray	10.41^a^	29.33	112.89^a^	46.05	75.94^c^	8.44	122.87^ab^	57.26	40.29^b^	36.72
Cur+ Flood	9.84^b^	22.24	76.93^e^	-0.52	93.33^ab^	33.27	81.32^d^	4.08	32.58^cd^	10.55
Cur + mix	8.08^c^	0.12	93.48^bc^	20.96	71.44^c^	2.01	101.68^c^	30.14	35.08^bcd^	19.04
BCA + Cur+ Spray	10.62^a^	31.93	117.73^a^	52.26	84.36^abc^	20.46	134.11^a^	71.65	48.66^a^	65.12
BCA + Cur+ Flood	9.93^b^	23.35	85.61^cd^	10.74	98.94^a^	41.28	86.91^d^	11.24	36.49^bc^	23.28
BCA + Cur+ mix	8.19^c^	1.74	100.65^b^	30.27	79.18^bc^	13.07	114.39^bc^	46.41	38.21^bc^	29.66
Control + BCA	9.80^b^	---	84.11^de^	---	73.69^c^	---	84.77^d^	---	37.47^bc^	---
Control	8.50^c^	---	77.29^de^	---	70.03^c^	---	78.13^d^	---	29.5^d^	---

Curcumin (Cur) application methods either by spraying (Cur+spray), flooding (Cur+Flood) or an alternating regimen of spraying and flooding (Cur + mix). These same application methods were also tested in combination with the biocontrol agent (BCA)

Statistically significant differences between means were indicated by different letters (s) according to Tukey’s multiple range test at α = 0.05

For instance, the manganese content was the highest with Curcumin application via flooding in the presence of BCA, (98.94ppm, + 41.28%), which indicates that the mineral-specific responses might differ depending on the application method. The alternation of spray and flood methods with or without BCA treatments showed a slight increase in most elements but were less effective than spray treatments, probably due to less efficient delivery or absorption dynamics.

### Effect of different Curcumin application methods combined with the biological control agent on the macroelements concentrations in pepper leaves

Table 4 shows the impact of various treatments on the concentration of essential macro-elements (N, P, K, Ca, Mg) in pepper leaves, along with a comparative efficacy analysis.

**Table 4:** Effect of Curcumin application methods combined with the biological control agent on nutrient elements in pepper leaves

Treatments	Concentration of elements in pepper leaves (%)									
	*N*	% Inc.	*P*	% Inc.	K	% Inc.	Ca	% Inc.	Mg	% Inc.
Cur+ Spray	3.33^b^	11.74	0.38^b^	100.00	3.24^cd^	3.51	1.52^a^	27.73	0.94^ab^	34.29
Cur+ Flood	3.07^bc^	3.02	0.24^c^	26.32	3.33^bc^	6.39	1.22^b^	2.52	0.75^de^	7.14
Cur + mix	4.09^a^	37.25	0.51^a^	168.42	4.45^a^	42.17	1.45^a^	21.85	0.89^bc^	27.14
BCA + Cur+ Spray	3.36^b^	12.75	0.39^b^	105.26	3.26^cd^	4.15	1.48^a^	24.37	0.98^a^	40.00
BCA + Cur+ Flood	3.09^bc^	3.69	0.25^c^	31.58	3.31^bcd^	5.75	1.24^b^	4.20	0.87^c^	24.29
BCA + Cur+ mix	4.30^a^	44.30	0.55^a^	189.47	3.47^b^	10.86	1.48^a^	24.37	0.93^abc^	32.86
Control + BCA	3.10^bc^	---	0.22^c^	---	3.19^cd^	---	1.23^b^	---	0.79^d^	---
Control	2.98^c^	---	0.19^c^	---	3.13^d^	---	1.19^b^	---	0.70^e^	---

Curcumin (Cur) application methods either by spraying (Cur+spray), flooding (Cur+Flood) or an alternating regimen of spraying and flooding (Cur + mix). These same application methods were also tested in combination with the biocontrol agent (BCA)

Statistically significant differences between means were indicated by different letters (s) according to Tukey’s multiple range test at α = 0.05

The figures indicate that nutrient uptake was substantially improved when Curcumin and bio-control agents (BCA) were applied, especially in the case of alternating application and foliar (spray) application methods. For instance, the nitrogen content varied between 2.98% (control) and 4.30% (The alternating application of spraying and flooding with Curcumin treatment in the presence of BCA). The alternating application of spraying and flooding with Curcumin treatment with or without BCA was far more effective than other treatments, with increases of 44.30 and 37.25%, respectively, thus, demonstrating the effectiveness of combined application strategies in enhancing nitrogen assimilation. The lowest N level among the different treatments was found under the application of Curcumin flood (3.07, 3.02% increase), thus, indicating that uptake efficiency might be reduced under this method. Phosphorus concentrations showed the most substantial changes of all nutrients. The alternating application of spraying and flooding with Curcumin treatment with or without BCA were the highest (0.55and 0.51%, respectively), thus, representing 189.47and 168.42% of increase, which is indicative of strong synergistic effects between Curcumin and BCA when they are combined and applied in the mixed form. The P content of the flood-based treatments was consistently at the lowest level, (0.24, 0.25%).

Each treatment raised potassium content when compared to the control, where the most considerable increase was obtained from the alternating method of spraying and flooding with Curcumin (4.45, 42.17% increase). Next, it was the same alternating application with the biological control agent (BCA) (3.47, 10.86% increase) that was leading in potassium content.

The calcium levels were greatly enhanced by the single application of Curcumin spray or the combination of Curcumin spray with a biological control agent (BCA), as well as the alternating spraying and flooding with Curcumin in the presence of BCA. The values of calcium concentration were from 1.45 to 1.52%, while the percentage of the increase ranged from 21.85 to 27.73. Without a doubt, these treatments can stimulate cell wall development and help pepper plants become more resistant to stress. On the other hand, the flood-based treatments had very slight to almost no effect on the Ca uptake, and their increase was as low as 2.52%.

Magnesium concentrations followed a similar trend to other nutrients, having their maxima in the Curcumin foliar spray and the alternating Curcumin spray and flood treatments (0.94–0.98%), with increase up to 40.00%. This may indicate a possible enhancement of chlorophyll synthesis. On the contrary, the Curcumin by flooding method showed the lowest magnesium concentrations and change, going down to only 7.14%. These results highlight the flood irrigation method as lesser contributing to magnesium bioavailability and uptake in plants.

### Influence of different Curcumin application methods on the macroelements, total carbohydrates, and capsaicin in pepper fruits

Data in (Table 5) illustrate the impact of different treatments on the concentrations of essential macronutrients (N, P, K), total carbohydrates, and capsaicin in the fruits of the pepper plant. The findings reveal that treatments caused significant differences in nutrient uptake as well as in the generation of secondary metabolites, which means that not only the treatment but also the way it was applied had an effect.

**Table 5:** Effect of Curcumin application methods combined with the biological control agent on key macronutrients (N, P, K), total carbohydrates, and capsaicin in pepper fruits

Treatments	Concentration of elements in fruits									
	*N*	% Inc.	*P*	% Inc.	K	% Inc.	%, Total Carbohydrate	% Inc.	Capsaicin	% Inc.
Cur+ Spray	2.41^a^	37.71	0.22^ab^	37.50	2.31^a^	11.06	0.53^ab^	35.90	103.19^b^	26.97
Cur+ Flood	1.81^c^	3.43	0.17^bc^	6.25	1.98^c^	-4.81	0.27^e^	-30.77	70.52^f^	-13.23
Cur + mix	2.31^ab^	32.00	0.21^abc^	31.25	2.36^a^	13.46	0.58^a^	48.72	102.69^b^	26.36
BCA + Cur+ Spray	2.44^a^	39.43	0.20^abc^	25.00	2.23^a^	12.98	0.55^a^	41.03	107.34^a^	32.08
BCA + Cur+ Flood	1.83^bc^	4.57	0.18^abc^	12.50	2.09^b^	0.48	0.30^de^	-23.08	76.44^e^	-5.94
BCA + Cur+ mix	2.37^a^	35.43	0.23^a^	43.75	2.39^a^	14.90	0.62^a^	58.97	106.30^a^	30.80
Control + BCA	2.09^abc^	---	0.20^abc^	---	2.15^b^	---	0.42^bc^	---	93.36^c^	---
Control	1.75^c^	---	0.16^c^	---	2.08^b^	---	0.39^cd^	---	81.27^d^	---

Curcumin (Cur) application methods either by spraying (Cur+spray), flooding (Cur+Flood) or an alternating regimen of spraying and flooding (Cur + mix). These same application methods were also tested in combination with the biocontrol agent (BCA)

Statistically significant differences between means were indicated by different letters (s) according to Tukey’s multiple range test at α = 0.05

Among all treatments, spraying Curcumin in the presence of BCA or without it showed the highest nitrogen concentrations (2.44 and 2.41%, respectively), representing a 39.43 and 37.71% of increase compared to the control. On the other hand, using Curcumin through flood without or with BCA had the least N levels (1.81 and 1. 83%, respectively), with insignificant increments, thus suggesting that nitrogen absorption or translocation might have been limited in flood irrigated plants. The alternating application of spraying and flooding with Curcumin and spraying with Curcumin only, also demonstrated enhanced phosphorus content (0.21– 0.22%), with percent of increase up to 37.50, indicating synergistic effects when Curcumin is combined with foliar application. The most significant result was the combination of spraying and flooding with Curcumin in the presence of BCA led to the highest P concentration (0.23%) hence a microbial inoculant’s positive role has been further proven for nutrient acquisition. On the other hand, a decrease in P content was observed in samples treated with Curcumin alone, with or without the BCA (0.17–0.18%).

The greatest potassium content was found in the potassium alternating application of spraying and flooding with Curcumin treatment (2.36%) and that in the presence of BCA was (2.39%), with percent of increase 13.46 and 14.90, respectively, compared to the control. The results obtained here are evidence that K uptake is improved by the use of mixed application methods (combining foliar and soil treatments). Potassium content of the plants treated with flood-Curcumin-application was decreased as evidenced by a negative percent of increase (-4.8%), which would be consistent with potassium being leached or poorly absorbed under this method. The greatest enhancement of the carbohydrate pool was seen with the combined spraying and flooding with Curcumin in the presence of BCA (0.62%) total carbohydrate content followed closely by alternating application without BCA (0.58%) with a 58.97% of increase, that of the first case. This situation may be due to increased photosynthetic activity and carbohydrate transport resulting from an improved nutrient supply. Lower carbohydrate levels have been observed when applying Curcumin Flood without or with BCA (–30.77% and − 23.08%), respectively, what could be the result of stress or lower metabolic activities in such conditions.

Significant variations in capsaicin contents were found among different treatments with the highest concentrations recorded in an experiment where Curcumin was sprayed in the presence of BCA (107.34 mg/g) and the alternating application of spraying and flooding with Curcumin treatment in the presence of BCA (106.30 mg/g), thereby making a 32.08 and 30.80% of increase, correspondingly. Hence this research points out one of the benefits that microbial bio-stimulants might have in raising the production of secondary metabolites in plants likely through nutrient availability or regulating the stress response pathways. On the contrary, the performance of applying Curcumin flood without or with BCA, as far as the percent of change, was negative (-13.23 and − 5.94), thus pushing the idea that capsaicin accumulation is least favored under flood.

## Discussion

The increasing occurrence of fungicide-resistant diseases and the highly toxic nature of the antifungal treatments currently in use are major global public health concerns. There is an urgent need to develop novel antifungal therapies with distinct target mechanisms. In this respect, naturally derived compounds have been advertised as potential antifungal agents because of their various biological activities, low toxicities, and wide availabilities [34]. Curcumin, a natural compound derived from turmeric (*Curcuma longa*), offers a biodegradable and effective plant-based antifungal option [35]. Curcumin exhibits potent inhibition against *Fusarium oxysporum*, its efficacy extends to other genera such as *Rhizoctonia* and *Alternaria* [17, 35, 36].

It has been common practice to use synthetic fungicides for a long time in order to control crop diseases. However, due to environmental and health issues, microbial biofungicides have become an eco-friendlier option. Nevertheless, the effect of microbial biofungicides is usually slower, their shelf life is shorter, and they are less accessible to farmers. To address these limitations, an integrated approach combining plant-based compounds with microbial biofungicides is recommended to balance effectiveness with sustainability [37].

The present study demonstrates the efficacy of Curcumin, both alone and in combination with biological control agents (BCA), in suppressing *Fusarium oxysporum* f. sp. *capsica*, promoting pepper plant growth and modulating key physiological and biochemical parameters. The antifungal assays revealed a clear dose-dependent inhibitory effect of Curcumin on radial fungal growth, with the highest tested concentration (3.0%) yielding a substantial 62.2% inhibition compared to the control. These findings align with earlier reports highlighting the multi-target antifungal mechanisms of Curcumin, including disrupting fungal cell membranes, inhibiting ergosterol synthesis, essential for maintaining fungal cell integrity, interfering with respiration and energy metabolism enzymes such as succinate dehydrogenase, and inducing oxidative stress within fungal cells. These multiple targets make it harder for fungi to develop resistance [17, 38, 39].

Modern irrigation methods such as drift and spray irrigation have evolved to not only deliver water efficiently but also incorporate plant treatments like fertilizers and pesticides directly through the irrigation systems, a practice known as fertigation or chemigation. These methods optimize the use of water and agrochemicals by targeting the root zone precisely, reducing waste and environmental impact. Advanced systems often include sensors and automation for real-time monitoring and control, enhancing both irrigation and treatment effectiveness. This integrated approach supports sustainable agriculture by improving crop health and conserving resources simultaneously [40].

Based on the results of greenhouse trails, when Curcumin applied in combination with the biological control agent *T. afroharzianum* (BCA), it notably enhanced plant growth parameters, disease severity reduction, and control efficacy in pepper plants. The foliar spray application of Curcumin alongside BCA resulted in the most pronounced increases in plant height (+ 51.9%), root length (+ 40.1%), fruit weight (+ 72.7%), and disease control efficacy (65.1%). Such synergistic interactions between botanical compounds and microbial BCAs are well documented, where Curcumin’s bioactivity complements the antagonistic effects of *Trichoderma* species against pathogens [41–43].

Integrating *Trichoderma* species with compatible agents such as fungicides, nanoparticles, beneficial microbes, elicitors, or organic amendments represents a cornerstone of effective, environmentally sustainable Fusarium wilt management. In the present study, the combination of *T. afroharzianum* with Curcumin achieved the most pronounced disease suppression in hot pepper, alongside substantial improvements in shoot biomass, mirroring findings where *T. virens* paired with sodium silicate enhanced growth parameters and reduced tomato Fusarium wilt severity. Biochemical analyzes further corroborated these benefits, revealing elevated peroxidase activity and phenolic compound accumulation in treated plants relative to untreated controls exhibiting maximum disease incidence. Collectively, these outcomes underscore the potential of Trichoderma-based integrations as viable, eco-friendly alternatives to synthetic fungicides, aligning with integrated disease management principles for soil-borne pathosystems [44].

Hormonal analysis revealed that Curcumin spray treatments elevated Gibberellic acid (GA3) levels, particularly when combined with BCA, suggesting a synergistic hormone-modulating effect that potentially contributes to improved growth vigor. In contrast, flooding applications of Curcumin reduce GA3 concentrations, which may explain observed reductions and growth parameters under this method. Similarly, Abscisic acid (ABA) levels were lowest in spray treatments, indicating alleviation of stress-related hormone accumulation. Activities of antioxidant enzymes Catalase (CAT) and Peroxidase (POD) followed consistent patterns, with the highest activities recorded in flood treatments, likely reflecting increased oxidative stress, while foliar spray applications corresponded with reduced enzymatic stress markers. The specific role of Curcumin was clearly explained by Zhang [45] which revealed that exogenous Curcumin application orchestrates a multifaceted defense strategy by simultaneously modulating ROS signaling, phytohormone dynamics, and secondary metabolite biosynthesis induced oxidative damage and improved plant resilience. Moreover, Curcumin influences plant metabolism by altering the synthesis of amino acids, fatty acids, and secondary metabolites, including nitrogen-containing compounds, terpenes and phenylpropanoids, all of which contribute cooperatively to the plant’s defense response.

Tofan [46] demonstrated that Curcumin treatment in sunflower plants exposed to abiotic stress significantly enhanced plant growth, increased total photosynthetic pigment content, stimulated the production of secondary metabolites, and boosted antioxidant enzyme activities, while simultaneously reducing the levels of oxidative stress markers.

Anas [47] mentioned that Curcumin significantly enhances the activity of key antioxidant enzymes, including including superoxide dismutase (SOD), catalase (CAT), and glutathione peroxidase (GPx). These enzymes serve as crucial components in the detoxification of reactive oxygen species (ROS), thereby preventing cellular damage and fostering improved stress resilience in both plants and humans. The increased enzyme activities observed upon Curcumin treatment contribute to their potent antioxidative and protective effects.

Micronutrient analysis of pepper leaves highlighted that foliar Curcumin application in the presence of BAC significantly enhanced concentrations of copper iron, zinc, and boron, indicating improved nutrient uptake possibly due to healthier roots or increased foliar absorption efficiency. This enhanced nutrient assimilation may underpin the improved growth and resistance observed. In contrast, Curcumin flooding application showed less favorable results, supporting the notion that application method critically influences bioavailability and efficacy. This is consistent with findings by Al-Ogaidi and Alwan [48], who demonstrated that foliar sprays of micronutrients resulted in higher absorption and yield compared to applications via irrigation water.

Macronutrient measurements further corroborated these findings; combined foliar and flooding applications with Curcumin and BCA notably increased nitrogen, phosphorus, potassium, calcium, and magnesium content in pepper leaves. Phosphorus exhibited especially marked increases, highlighting possible synergistic effects of Curcumin and microbial inoculants in facilitating nutrient acquisition and metabolism, Kumari [49], demonstrated that BCA act as biofertilizers, soil improvers, growth regulators, stress relievers, and biocontrol agents.

Capsaicin biosynthesis is controlled by complex genetic and environmental factors, including the expression of key biosynthesis genes regulated by various transcription factors (e.g., MYB31, WRKY9) [50] and enzymatic activities within the phenylpropanoid and branched-chain fatty acid pathways [51]. Secondary metabolites like capsaicin are sensitive to external stress signals, and foliar application tends to stimulate these pathways effectively, enhancing enzyme activities (such as phenylalanine ammonia-lyase, cinnamic acid 4-hydroxylase, capsaicin synthase) and increasing phenolic compounds, ultimately promoting capsaicin accumulation [52].

In contrast, flooding or excessive soil application of Curcumin may interfere with root oxygen levels or induce stress responses that divert metabolic resources away from capsaicinoid synthesis or increase the activity of degradative enzymes such as peroxidases, which catalyze capsaicin oxidation, reducing its concentration. Thus, the delivery method profoundly influences capsaicin levels by differentially impacting metabolic regulation and stress response signaling.

In summary, the reduction of capsaicin following flooding Curcumin applications highlights the importance of delivery method in modulating capsaicinoid biosynthesis and plant health, supporting the observation that foliar Curcumin treatments are more effective at enhancing content. Finally, the concentration of capsaicin, a key bioactive secondary metabolite in pepper fruits was significantly elevated by foliar Curcumin treatments with or without BCA, consistent with literature indicating microbial and natural product elicitation of secondary metabolism related to stress tolerance and plant defense. Flooding Curcumin applications, however, were associated with reductions in capsaicin, underscoring again the importance of delivery method.

## Conclusion

The data collectively demonstrate that Curcumin^’^s antifungal and plant growth-promoting effects are significantly enhanced when combined with the biocontrol agent *T. afroharzianum*. Foliar spraying emerges as the most effective application method, optimizing growth, disease resistance, hormonal regulation, and nutrient uptake, while flood irrigation with Curcumin displays comparatively limited benefits. These findings support the strategic integration of botanical bioactives and microbial biocontrol agents in sustainable pepper cultivation systems.

## Data Availability

The datasets used and/or analysed during the current study are available from the corresponding author on reasonable request.
